# Outcomes of Complex Sleeve Resections by Video-Assisted Thoracic Surgery: Single-Centre Study

**DOI:** 10.1093/icvts/ivag225

**Published:** 2026-08-17

**Authors:** Volkan Erdoğu, Melike Ülker, Ayşegül Çiftçi, Merve Ekinci Fidan, Nisa Yıldız, Çiğdem Obuz, Salih Bilen, Esra Gözügül, Tuğçe Barça Şeker, Muzaffer Metin

**Affiliations:** Department of Thoracic Surgery, Yedikule Chest Diseases and Thoracic Surgery Training and Research Hospital, Istanbul, Turkey; Department of Thoracic Surgery, Yedikule Chest Diseases and Thoracic Surgery Training and Research Hospital, Istanbul, Turkey; Department of Thoracic Surgery, Yedikule Chest Diseases and Thoracic Surgery Training and Research Hospital, Istanbul, Turkey; Department of Thoracic Surgery, Yedikule Chest Diseases and Thoracic Surgery Training and Research Hospital, Istanbul, Turkey; Department of Thoracic Surgery, Yedikule Chest Diseases and Thoracic Surgery Training and Research Hospital, Istanbul, Turkey; Department of Thoracic Surgery, Yedikule Chest Diseases and Thoracic Surgery Training and Research Hospital, Istanbul, Turkey; Department of Thoracic Surgery, Yedikule Chest Diseases and Thoracic Surgery Training and Research Hospital, Istanbul, Turkey; Department of Anesthesia and Reanimation, Yedikule Chest Diseases and Thoracic Surgery Training and Research Hospital, Istanbul, Turkey; Department of Anesthesia and Reanimation, Yedikule Chest Diseases and Thoracic Surgery Training and Research Hospital, Istanbul, Turkey; Department of Thoracic Surgery, Yedikule Chest Diseases and Thoracic Surgery Training and Research Hospital, Istanbul, Turkey

**Keywords:** VATS, sleeve resection, carinal reconstruction, bronchovascular sleeve, minimally invasive thoracic surgery

## Abstract

Complex sleeve resections performed by video-assisted thoracic surgery (VATS) are technically demanding procedures requiring advanced surgical experience. We retrospectively evaluated 21 complex sleeve resections performed by VATS between January 2018 and October 2024. Complex sleeve resections included carinal sleeve pneumonectomy, isolated carinal reconstruction, bronchovascular sleeve lobectomy, vascular sleeve resections, sleeve lingulectomy, distal tracheal reconstruction, and segmental bronchial sleeve resections. Patient selection was based on tumour location, vascular and airway invasion, radiologic resectability, and the feasibility of achieving R0 resection through a minimally invasive approach. The mean operative time was 195 ± 70.2 min, and the average blood loss was 340 ± 135.2 mL. Mean postoperative hospital stay was 5.5 ± 1.74 days. Early postoperative morbidity occurred in 4 patients (19%), and postoperative mortality occurred in 1 patient (4.7%). R0 resection was achieved in all malignant cases. In experienced centres with advanced minimally invasive thoracic surgery expertise, complex sleeve resections can be safely performed by VATS with acceptable perioperative morbidity and mortality.

## GRAPHICAL ABSTRACT

**Figure:**
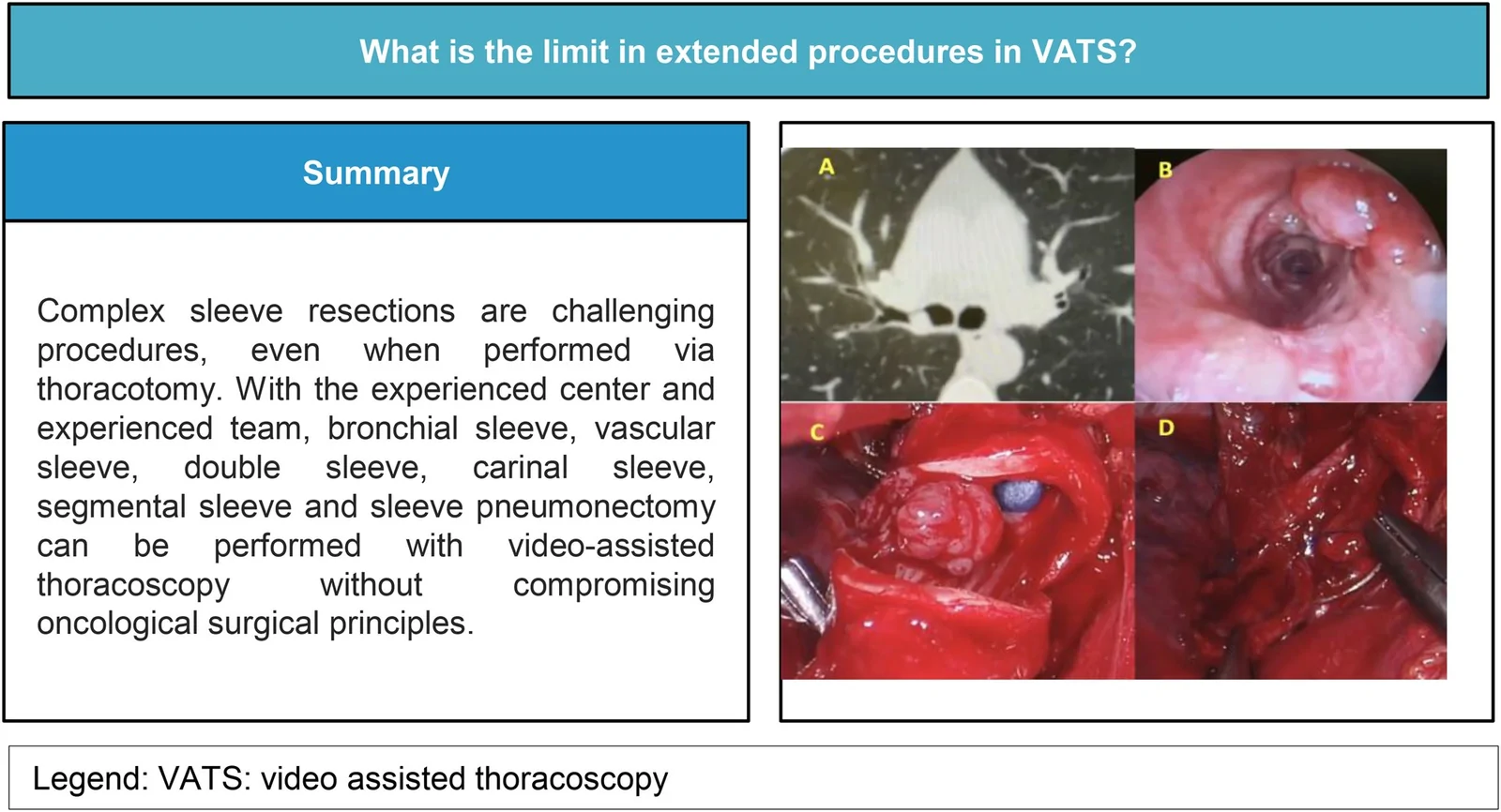

## INTRODUCTION

Video-assisted thoracic surgery (VATS) has become widely used in thoracic surgery over the last decades.^1^^,^^2^ Initially utilized for diagnostic procedures and wedge resections, increasing surgical experience has enabled the use of VATS for complex anatomical lung resections and airway reconstruction procedures.^2^^,^^3^ Sleeve resections are important parenchyma-sparing procedures in both malignant and benign diseases.^4^ With advances in minimally invasive surgery, increasingly complex sleeve resections, including bronchovascular and carinal reconstructions, have become feasible in selected patients.^2^^,^^3^^,^^5^

Complex sleeve resections are technically demanding procedures that require advanced surgical experience and careful patient selection. Although there is no universally accepted definition, procedures involving carinal reconstruction, vascular sleeve resections, bronchovascular resections, and segmental airway reconstructions are generally considered complex sleeve procedures.^3^^,^^5^

The aim of this study was to present the perioperative and early postoperative outcomes of complex sleeve resections performed by VATS in a high-volume thoracic surgery centre and to summarize important technical considerations.

## MATERIALS AND METHODS

A total of 21 complex sleeve resections performed by VATS between January 2018 and October 2024 were retrospectively evaluated. Complex sleeve procedures included carinal sleeve pneumonectomy, isolated carinal reconstruction, bronchovascular sleeve lobectomy, isolated vascular sleeve resections, distal tracheal reconstruction, sleeve lingulectomy, and segmental bronchial sleeve resections.

Two carinal sleeve pneumonectomy cases and one bronchovascular sleeve case converted to thoracotomy due to technical difficulty were excluded from the study.

All procedures were performed under general anaesthesia with selective intubation in the lateral decubitus position. Double-lumen intubation, endobronchial blocker, or high-frequency jet ventilation techniques were used according to operative requirements.

At the time of the study period, 6 thoracic surgeons were working in our department. However, all complex sleeve resections included in this series were performed by 2 surgeons experienced in advanced VATS airway surgery.

Most procedures were performed using a biportal approach (66%). Surgical indication was determined according to preoperative radiologic findings and perioperative assessment.

Patient selection for VATS complex sleeve resection was based on tumour location, extent of airway and vascular invasion, radiologic resectability, and the anticipated feasibility of achieving R0 resection through a minimally invasive approach. Patients with extensive invasion requiring complex reconstruction or cases considered unsuitable for minimally invasive exposure were preferentially managed with thoracotomy. The decision for conversion was related to technical difficulties during surgery; no patient required conversion because of bleeding.

The 8th edition of the TNM classification was used for tumour staging.

## SURGICAL TECHNIQUE

Systematic mediastinal lymph node dissection was routinely performed in malignant cases. Airway anastomoses were generally performed using continuous 3/0 non-absorbable sutures after frozen-section confirmation of negative margins.

In malignant cases, mediastinal lymph node dissection was generally performed before completion of the bronchial anastomosis in order to improve exposure and facilitate a tension-free reconstruction.

Particularly in left-sided sleeve resections, looping and suspending the pulmonary artery provided improved exposure during bronchial reconstruction.

In our experience, adequate hilar release, careful lymph node dissection, and minimizing tension on the anastomosis are key technical principles for preventing anastomotic complications.

The procedures included carinal sleeve pneumonectomy (*n* = 6), isolated carinal resection and reconstruction (*n* = 2), bronchovascular sleeve lobectomy (*n* = 1), isolated vascular sleeve resections (*n* = 3), sleeve lingulectomy (*n* = 2), distal tracheal resection and reconstruction (*n* = 1), and segmental bronchial sleeve resections (*n* = 3).

## RESULTS

Of the 21 patients, 15 were male (71%), and the mean age was 58.3 ± 11.74 years. Nineteen cases were malignant, including NSCLC, carcinoid tumours, glomus tumour, SCC, adenocarcinoma, pleomorphic carcinoma, and sarcoma. R0 resection was achieved in all malignant cases.

Patient demographics, histopathological diagnoses, and types of surgical procedures are summarized in **Table 1**.

**Table 1.** Characteristic of Patients, Diagnosis, and Type of Surgery

Patients	Gender	Age	Diagnosis	Type of Surgery
1	M	46	Globus tumour localized in the right main bronchus	Right main bronchus resection and sleeve reconstruction (Figure: Graphical abstract)
2	M	63	Left upper lobe centrally located SCC T1bN1 (Stage IIB)	Left sleeve upper lobectomy and arterioplasty
3	M	62	Left upper lobe centrally located Adenocarcinoma-T2aN2 (Stage IIIA)	Left sleeve upper lobectomy and arterioplasty
4	M	56	Left upper lobe centrally located Adenocarcinoma-T2aN1 (Stage IIB)	Left sleeve upper lobectomy and arterioplasty
5	F	58	Left upper lobe centrally located Pleomorphic carcinoma-T3N0(Stage IIB)	Left bronchovascular sleeve upper lobectomy
6	M	23	Carinal located SCC	Right VATS carina resection and reconstruction (Neocarina)
7	F	34	Carinal located Follicular dendritic cell sarcoma	Right VATS carina resection and reconstruction (Neocarina)
8	M	61	Right upper lobe centrally located Adenocarcinoma-T3N1 (Stage IIIA)	Right carinal sleeve pneumonectomy
9	M	67	Right upper lobe centrally located SCC-T2aN2 (Stage IIIA)	Right carinal sleeve pneumonectomy after neoadjuvant chemotherapy
10	M	69	Right upper lobe centrally located SCC-T2aN0 (Stage IB)	Right carinal sleeve pneumonectomy after neoadjuvant chemo-immunotherapy
11	M	67	Right upper lobe centrally located SCC-T4N2 (Stage IIIB)	Right carinal sleeve pneumonectomy
12	M	47	Right upper lobe centrally located Adenocarcinoma-T4N2 (Stage IIIB) Emergency surgery due to haemoptysis	Right carinal sleeve pneumonectomy and tangential resection of superior vena cava
13	M	64	Right upper lobe centrally located Adenocarcinoma-T2bN0 (Stage IIB)	Right carinal sleeve pneumonectomy
14	F	53	Bronchiectasis in the left lower lobe and lingula	Left lower lobectomy and sleeve lingulectomy
15	F	43	Typical carcinoid tumour at the entrance of the lingula	Left sleeve lingulectomy
16	M	26	Typical carcinoid tumour in the right intermediate bronchus	Right intermediate bronchus segmental resection and sleeve reconstruction
17	M	60	Typical carcinoid tumour in the right intermediate bronchus	Right intermediate bronchus segmental resection and sleeve reconstruction
18	F	57	Postentubation distal tracheal stenosis	Distal trachea resection and sleeve reconstruction
19	F	61	Left upper lobe centrally located Adenocarcinoma-T2N0 (Stage IIA)	Left upper vascular sleeve lobectomy
20	M	63	Left upper lobe centrally located Adenocarcinoma-T0N0 (Stage 0)	Left upper vascular sleeve lobectomy after neoadjuvant chemotherapy
21	M	67	Left upper lobe centrally located SCC-T2bN0 (Stage IIA)	Left upper vascular sleeve lobectomy after neoadjuvant chemo-immunotherapy

The mean operative time was 195 ± 70.2 minutes, and the average blood loss was 340 ± 135.2 mL. Mean postoperative hospitalization was 5.5 ± 1.74 days.

Early postoperative morbidity occurred in 4 patients (19%). Two patients developed atrial fibrillation after carinal sleeve pneumonectomy. One patient developed wound infection after bronchovascular sleeve lobectomy performed following neoadjuvant therapy. One patient developed bronchopleural fistula and Acute Respiratory Distress Syndrome (ARDS) after carinal sleeve pneumonectomy and died on postoperative day 6.

Perioperative outcomes, postoperative complications, and mortality data are summarized in **Table 2**. Overall postoperative mortality was 4.7%.

**Table 2.** Perioperative and Postoperative Process

Patients	Surgery	Number of port	Operation time (minutes)	Perioperative bleeding (mL)	Hospitalization (day)	Complication
1	Right main bronchus resection and sleeve reconstruction	2	150	200	4	0
2	Left sleeve upper lobectomy and arterioplasty	2	240	400	4	0
3	Left sleeve upper lobectomy and arterioplasty	3	270	500	4	0
4	Left sleeve upper lobectomy and arterioplasty	2	230	400	3	0
5	Left bronchovascular sleeve upper lobectomy	1	300	350	4	Atrial fibrillation
6	Right VATS carina resection and reconstruction (Neocarina)	3	150	300	4	0
7	Right VATS carina resection and reconstruction (Neocarina)	2	220	0-300 mL	7	0
8	Right carinal sleeve pneumonectomy	2	300	400	ex	ARDS-BPF
9	Right carinal sleeve pneumonectomy	2	300	600	7	0
10	Right carinal sleeve pneumonectomy	1	270	500	7	Atrial fibrillation
11	Right carinal sleeve pneumonectomy	2	270	850	7	0
12	Right carinal sleeve pneumonectomy	1	280	400	7	0
13	Right carinal sleeve pneumonectomy	2	260	300-500	7	0
14	Left sleeve lingulectomy	2	240	600	4	0
15	Left sleeve lingulectomy	2	180	500-700	3	0
16	Sağ intermedier bronş segmental rezeksiyonu ve sleeve rekonstrüksiyonu	2	160	300	3	0
17	Right intermediate bronchus segmental resection and sleeve reconstruction	1	180	300	3	0
18	Distal trachea resection and sleeve reconstruction	3	210	300	7	Wound infection
19	Left upper vascular sleeve lobectomy	2	310	1200	4	0
20	Left upper vascular sleeve lobectomy	3	220	500-700	3	0
21	Left upper vascular sleeve lobectomy	2	250	450	4	0

Abbreviations: ARDS, Acute Respiratory Distress Syndrome; BPF, bronchopleural fistula.

## DISCUSSION

Bronchial sleeve resections preserve lung parenchyma and may avoid pneumonectomy-associated morbidity and mortality.^4^ With increasing experience in minimally invasive thoracic surgery, VATS sleeve resections have become feasible in experienced centres.^2^^,^^3^^,^^5^ However, complex sleeve resections remain technically demanding procedures requiring advanced surgical expertise.

Previous studies have emphasized the importance of surgical experience and learning curve in advanced VATS airway surgery.^6–8^ Our institution is a high-volume referral centre for thoracic oncology and lung cancer surgery, performing approximately 800 VATS resections and 25 sleeve resections annually. We believe that institutional experience and surgical team continuity are critical factors influencing perioperative outcomes in complex sleeve resections.

In our opinion, these procedures should initially be concentrated in experienced surgical teams and high-volume centres before wider dissemination to other units.

Recent VATS sleeve resection series have reported acceptable morbidity and mortality rates comparable to thoracotomy.^3^^,^^5^^,^^6^ Carinal resections remain among the most technically challenging thoracic surgical procedures.^4^^,^^5^ In our series, bronchopleural fistula developed in one patient following postoperative ARDS and reintubation after carinal sleeve pneumonectomy.

The development of minimally invasive vascular reconstruction techniques and airway management strategies has expanded the indications for VATS complex sleeve procedures.^2^^,^^5^^,^^7^ Careful hilar release, minimizing anastomotic tension, and optimal exposure remain critical technical principles.^9^^,^^10^

The main limitation of our study is its retrospective single-centre design. The study mainly focused on perioperative and early postoperative outcomes. Long-term outcomes, including 90-day mortality and readmission analysis, were beyond the scope of this retrospective evaluation.

The relatively limited number of patients and the heterogeneity of the included procedures represent important limitations and reduce the generalizability of the conclusions. Future multicentre studies with larger cohorts may provide more robust evidence regarding the reproducibility and oncologic validity of VATS complex sleeve resections.

In conclusion, complex sleeve resections can be safely performed by VATS in experienced centres with acceptable morbidity and mortality rates.

## Supplementary Material

ivag225_Supplementary_Data

## Data Availability

“Statistical Package for the Social Sciences” (SPSS) Statistics Version 26 and propensity score program were used. The data in SPSS format can be sent upon request.
