# Supplementary material for: Outcomes of Complex Sleeve Resections by Video-Assisted Thoracic Surgery: Single-Centre Study
Source: Interdiscip Cardiovasc Thorac Surg. 2026 Aug 17;41(9):ivag225. doi: 10.1093/icvts/ivag225 (PMC13581395; doi:10.1093/icvts/ivag225)
Supplement: Supplementary file 1 [file ivag225_supplementary_data.zip › Supplementary Figure Legends.docx]

**Figure Legends**

**Figure 1.** Pulmonary arterioplasty

**Figure 2.** Bronchovascular sleeve lobectomy

**Figure 3.** Isolated carinal reconstruction
